# Degrees and Diplomas

**Published:** 1902-09-06

**Authors:** 


### Degrees and Diplomas.

The changes which are made from time to time in the course of study required for certain medical qualifications render it essential that the student should fully and carefully inquire into the whole matter before embarking upon his career. It is unfortunately the fact that, owing to the number of portals to the profession, the variety of qualifications which are, so to say, in the market, and the lack of co-operation between the several qualifying bodies, much time and effort may be wasted unless the student starts properly from the very beginning. This warning more especially applies to the matter of obtaining a degree, for it not infrequently happens that a student who has already advanced a long way towards his diploma finds that, in consequence of having neglected to matriculate at a university, it is impossible for him to obtain a medical degree without going back to elementary or even school-boy subjects. Some, when they make this discovery, face the ordeal, and at great waste of time and money, commence afresh; but to many this is impossible, and much to their chagrin and often greatly to their monetary loss, they have to put up with a diploma instead of a degree.

### The Penalties of Failure.

On the other hand, it may happen that a youth's ambition may soar too high and land him in serious difficulties, for if the money, and therefore the time at his disposal, be strictly limited, repeated efforts to get through the early stages of the higher qualifications may involve serious risk of his missing his qualification altogether, for failure at an examination may make many months of difference in the date at which the student becomes self-supporting. It must always be remembered that a medical education leads to nothing unless it ends in a qualification, and that it is no use a man spending all his money struggling with, to him, impossible examinations. Far better either turn to something else at once, or putting one's ambition in one's pocket accept such a diploma as may be obtainable.

The General Medical Council is the body appointed by law to control medical education and registration in the United Kingdom. It must be borne in mind, however, that although the rules legally laid down by the General Medical Council must in all cases be followed by the qualifying bodies, they refer merely to the minimum of instruction and the minimum of examination which is permitted. Most of the qualifying bodies grant diplomas or degrees, the requirements for which go far beyond the minimum so fixed, and these are generally spoken of as the "higher qualifications."

### The Choice of a Qualification.

In the choice of a qualification much depends upon the kind of practice which the student proposes to take up. It is herein that the great difficulty lies, for very few students, at the commencement of their curriculum, have the slightest idea in what direction their future life may drift, and this being so we can but advise those who have the opportunity to take a degree. If a student looks forward to a career as a consulting physician he must not only obtain a degree in medicine from a recognised university, but he must become a member of one of the Royal Colleges of Physicians, that of London, Edinburgh, or Dublin, according to the part of the United Kingdom in which it is his intention to settle. On the other hand, if it be surgery to which he desires to devote himself, he must obtain the Fellowship of one of the Royal Colleges of Surgeons; while so curiously is work distributed among the various branches of the profession that if a man should wish to obtain a good position in London in gynæcology, he would do well not only to obtain a university degree in medicine, but also to become a Fellow of the Royal College of Surgeons in England—for gynæcology is surgery, yet by a freak of circumstances it often falls into the hands of a so-called "obstetric physician," and some hospitals would hesitate to appoint a "physician" without a degree.

### General Practitioners.

For general practice any diploma or degree *will do*; it is enough to be on the Register, but the advantages of possessing a degree are becoming every year more and more manifest. With increasing competition anything which, by hint or innuendo, can be twisted into a slur upon his fitness or his qualifications becomes a serious disadvantage to a practitioner. Even if there be not at hand some kind professional "brother" to point out that the mere "diploma man" is "not a real doctor you know," there is the constant and reiterated annoyance of being continually addressed by a title which one knows one does not possess—a pin-prick, no doubt, but one which by repetition becomes extremely galling. Thus as "all roads lead to Rome," so all kinds of reasons point to the desirability of obtaining a degree in medicine if one would obtain for oneself a good position as a practitioner, and, what is still more important, freedom to take up any form of practice in whatever direction an opening may occur.

### London as a Place of Study.

If then a degree be aimed at, the question Where to take it? becomes a very important one, for this must decide to some extent the place of study. The manifold advantages of London as a place in which to go through the practical clinical portion of the medical curriculum are so great that we would urge all those who are able to do so to arrange that at least the second half of their period of study should be passed there. With this object the student may matriculate at and obtain the degree of the University of London. For those who feel able to pass fairly stiff examinations this is, we think, the best course. Another plan is to do the first half of the course at one of the provincial universities—Cambridge or Durham, or even Oxford if time is no object—then to come to London for the rest of the time, and finally to obtain the degree of the university at which the student began his studies. This is a plan which is often adopted, and has certain definite advantages. There is still a certain prestige about the Cambridge degree and perhaps more still in regard to that of Oxford, with the drawback in the latter case however that at Oxford a degree in Arts must be obtained.

### Other Universities.

In those cases in which it is determined not to study in London the choice of a university will probably be decided by various considerations which will be personal to the individual. Locality may draw people from the Midlands to Birmingham, from Lancashire and Yorkshire to the Victoria, from the Newcastle district to Durham, and from the various parts of Scotland and Ireland to Edinburgh, St. Andrews, Aberdeen, Glasgow, Dublin, or the various schools attached to the Royal University of Ireland, as the case may be.

### The Scottish Universities.

The Universities of Edinburgh and Aberdeen, however, and especially that of Edinburgh, must not be regarded from a merely local point of view, for to them men from all parts of the world flock to partake of the admirable system of medical instruction which there prevails. The mode of teaching in Scotland is somewhat different from that which obtains in most English schools, and report has it that the course can be gone through and the degree obtained there upon much cheaper terms than is the case in England. It is probable, however, that this is largely due to readiness of the Scottish student to live in a less expensive manner than is commonly adopted by students in England. Those, then, whose means are small should carefully consider the Universities of Scotland. It is always important that a student should feel on terms of equality with his fellows, and it is far easier and more pleasant to live frugally where others are doing the same, than to be the odd man in a crowd well supplied with the good things of this world.

### The Conjoint.

If, however, circumstances should combine to make it impossible to obtain a university degree, we have no hesitation in saying that the proper qualification to aim at is the English Conjoint, that is the L.R.C.P., M.R.C.S. granted by the Royal College of Physicians of London and the Royal College of Surgeons of England. This qualification serves as a stepping stone to the higher diplomas of both bodies, qualifications which are essential for nearly all good hospital appointments in London, and is therefore one which nearly all good men take even though many obtain university degrees as well.

Our advice then to the student who is not tied by local circumstances is—London, if possible ; if not, Cambridge or Durham for the first part of the curriculum, doing the hospital work in London ; and if not this, then Scotland, always endeavouring, whatever the school selected, to come out at the end of the course the possessor of a degree and the "Conjoint." If this, which is the best plan, cannot be followed, then either the Conjoint or a degree may be taken separately ; or, failing that, the student may become a Licentiate of the Society of Apothecaries, which at least will place him on the Register and enable him to practise. Once on the Register all are alike in the eyes of the law, whatever their qualifications. But they are not all alike in the eyes of the public nor in those of the profession, and thus it is an immense advantage to start life with a good degree.

